# The tomato *WV* gene encoding a thioredoxin protein is essential for chloroplast development at low temperature and high light intensity

**DOI:** 10.1186/s12870-019-1829-4

**Published:** 2019-06-20

**Authors:** Shenghua Gao, Wenjing Gao, Xiaoli Liao, Cheng Xiong, Gang Yu, Qihong Yang, Changxian Yang, Zhibiao Ye

**Affiliations:** 1 Key Laboratory of Horticultural Plant Biology (Ministry of Education), Huazhong Agricultural University, Wuhan, 430070 Hubei China; 2 Hubei Key Laboratory of Vegetable Germplasm Enhancement and Genetic Improvement, Cash Crops Research Institute, Hubei Academy of Agricultural Sciences, Wuhan, 430070 Hubei China

**Keywords:** Tomato, *Wv*, Leaf color, Chloroplast, Map-based cloning

## Abstract

**Background:**

Chloroplast biogenesis, a complex process in higher plants, is the key to photoautotrophic growth in plants. White virescent (*wv*) mutants have been used to unfold the molecular mechanisms underlying the regulation of chloroplast development and chloroplast gene expression in plants. However, most of genes controlling white virescent phenotype still remain unknown.

**Results:**

In this study, we identified a temperature- and light intensity-sensitive mutant, named as *wv*. The content of chlorophyll was dramatically decreased in the immature leaves of *wv* mutant under the conditions of low temperature and high-light intensity. TEM observation showed that the chloroplasts in the young leaves of *wv* mutant lacked an organized thylakoid membrane, whereas crescent-shaped chloroplasts with well-developed stromal and stacked grana thylakoids in the mature leaves were developed. Immunoblot analyses suggested that proteins of photosynthetic complexes were decreased substantially in *wv* mutants. Based on map-based cloning and transgenic analysis, we determined that the *wv* phenotype was caused by single base mutation in the first intron of *WV* gene, which encoded a thioredoxin protein with 365 amino acids. qRT-PCR analysis revealed that the expression of *WV* gene was significantly down-regulated in *wv* mutant. In addition, knockdown of *WV* gene through RNAi also resulted in white virescent young leaves, suggesting that the mutation possibly blocks the differentiation of chloroplasts through inhibiting the expression of *WV* gene. Furthermore, the expression of *WV* peaked in apical buds and gradually decreased along with the developmental stage, which was consistent with the *wv* mutant phenotype. Expression analysis of chloroplast-encoded genes by qRT-PCR showed that the *wv* mutation affected the expression pattern of chloroplast-encoded PEP dependent genes.

**Conclusion:**

Our results suggested that *wv* mutant was sensitive to low temperature and light intensity. *WV* gene was essential for chloroplast differentiation. A single base mutation in the first intron resulted in down-regulation of *WV* gene expression, which inhibited the expression of chloroplast-encoded genes, thereby blocking chloroplast formation and chlorophyll synthesis.

**Electronic supplementary material:**

The online version of this article (10.1186/s12870-019-1829-4) contains supplementary material, which is available to authorized users.

## Background

Chloroplasts are important cellular organelles of photoautotrophic eukaryotes, which differentiate from plastids in plant cells [1]. Chloroplasts are coordinately controlled by the proteins encoded in both plastid and nuclear genomes [2]. These proteins play important roles in protein translation, foldingand transport during chloroplast development [3]. Chloroplast development is tightly coupled with the leaf cell division and elongation. The later developmental process requires illumination, including chlorophyll accumulation and construction of the photosynthetic machinery [4]. Most of photosynthesis and some metabolic processes occurred in chloroplasts, such as synthesis of amino acids, fatty acids, carotenoids, vitamins, and a range of specialized metabolites [5].

Chlorophyll is critical for photosynthesis, which can capture light energy and drive photosynthetic reactions [6]. Chlorophyll biosynthesis is cooperatively regulated by 16 enzymes in *Arabidopsis* [7]. Glutamyl tRNA reductases (GluTR) encoded by *HEMA1*, *HEMA2*, and *HEMA3*, are important limiting factors of chlorophyll synthesis [8]. 5-aminolevulinic acid (ALA) is converted into uroporphyrinogen III and then oxidized into Proto by protoporphyrinogen oxidase. Three enzymes, including MgProtoMe cyclase, NADPH-Pchlide oxidoreductase, and Chl synthase, catalyze Proto into chlorophyll [9]. Block of each step can cause changes in chlorophyll content and leaf color. Different mutations caused diverse leaf colors, like albino, yellow, chlorina, light green, dark green, etc. In addition, leaf color patterns are tremendously varied, such as mono-colored, spotted, striped, blotched, zebra, and variegated [10]. Recently, many other leaf color mutants have been reported in rice [11, 12], wheat [13], maize [14], and foxtail millet [15].

Nowadays, many genes involved in chlorophyll biosynthesis or chloroplast formation have been characterized in many plants [7, 16–18]. *YGL1*, encoding a chlorophyll synthase, functions in the esterification of chlorophyllide and phytol in rice [19]. *NYC* encodes a chlorophyll b reductase, the mutation of which impaired chlorophyll degradation during senescence [20]. Chloroplast formation is essential for chlorophyll biosynthesis. Therefore, mutations in many genes required for chloroplast formation also caused leaf color changes. *AtSIG6*, a nuclear-encoded sigma factor in *Arabidopsis*, is critical for light-dependent chloroplast development. *AtSIG6* null mutant exhibited pale green phenotype in cotyledons [21]. EMB1303, a chloroplast-localized protein, controls chloroplast development in *Arabidopsis*. The *emb1303–1* mutant displayed albino rosette leaves [16]. In addition, overexpression of a RanBP2-type zinc finger transcription factor, *SlRBZ*, possibly impaired the biosynthesis of chlorophyll and resulted in chlorosis phenotype through blocking chloroplast development in tomato [22].

White or yellow virescent leaf mutants widely exist in nature, which have accelerated the characterization of molecular mechanisms underlying chlorophyll synthesis and chloroplast development. *Chlorina-1* mutant exhibited yellowish-green leaf phenotype under normal growth conditions at seedling stage, and then turned into normal green [23]. *virescent-2* mutant developed chlorotic leaves at restrictive temperature (20 °C), whereas developed green leaves at permissive temperature (30 °C) [24]. Two rice temperature-conditional mutants, *virescent3* (*v3*) and *stripe1* (*st1*), produced bleached leaves at a constant temperature of 20 °C or 30 °C, and green leaves under diurnal 30 °C/20 °C conditions [25]. In addition, one mutation in *NUS1* gene caused chlorotic rice leaves under low-temperature conditions [26]. Tu, a chloroplast protein synthesis elongation factor, was involved in chloroplast development. Loss-of-function mutation in *Tu* resulted in green-revertible albino phenotype in rice [27].

In this study, we identified a chlorophyll deficient mutant, named as *white virescent* (*wv*), which showed white virescent apical buds and immature leaves. We developed insertion-deletion (InDel) markers for genotyping of an F_2_ segregating population. *wv* was delimited in an approximate 94 kb region, which contained 18 putative candidates. Transgenic analysis indicated that the *wv* phenotype was caused by a single base mutation in the first intron of *WV* gene, which encodes a thioredoxin protein of 365 amino acids. qRT-PCR analysis and GUS activity revealed that the expression level of *WV* peaked in apical buds, and gradually decreased along with the developmental stage. Knockdown of *WV* gene by RNAi also caused white virescent young leaves. We thus speculate that the single base mutation in *WV* gene possibly blocked chloroplast differentiation by inhibiting the transcription of *WV* gene.

## Results

### *wv* mutant exhibits a thermo-sensitive phenotype

**Fig. 1:**
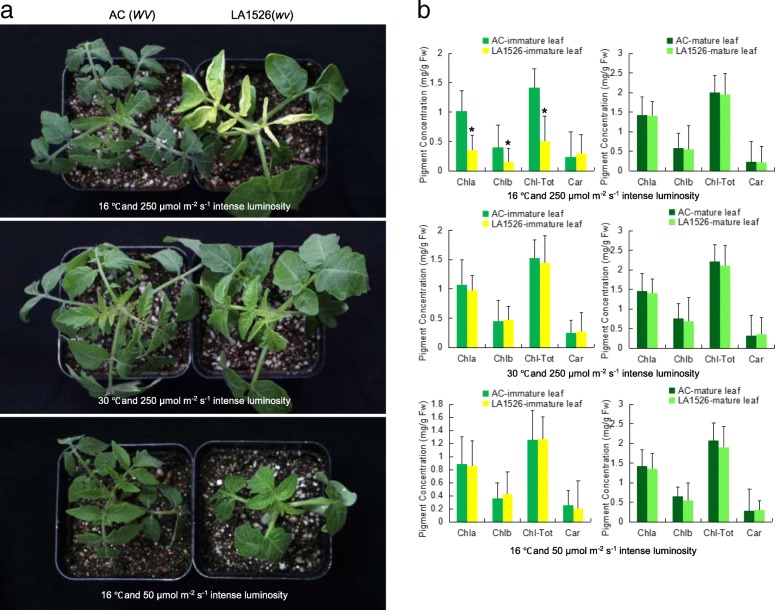
Phenotypic analysis of *wv* mutant LA1526. **a** Plants at different temperature and intense luminosity in the greenhouse. **b** The pigments contents in immature and mature leaves of *wv* mutants and AC plants at different temperature and intense luminosity. Chla, Chlorophyll a; Chlb, chlorophyll b; Chl-Tot, total chlorophyll; Car, carotenoid. Values are mean ± SD of three biological replicates. Student’s t test was performed on the raw data; asterisk indicates statistical significance at *P* < 0.01

*wv* mutant LA1526 displayed white/yellow leaves at 16 °C compared with AC plants (Fig. 1a). The yellow leaves gradually became virescent with temperature rising and exhibited green leaves at 30 °C. In addition, *wv* leaves gradually turned green from top to base (Fig. 1b). Interestingly, LA1526 showed the same leaf color as AC plants at 16 °C and low-light intensity of 50 μmol m^− 2^ s^− 1^ (Fig. 1c). These results suggested that *wv* mutant LA1526 may be sensitive to temperature and light. We further measured chlorophyll content and found that chlorophyll a, chlorophyll b and total chlorophyll drastically decreased in the immature leaves of LA1526 compared to AC plants at 16 °C and high-light intensity (250 μmol m^− 2^ s^− 1^) (Fig. 1d). However, both LA1526 and AC plants showed similar chlorophyll content when growing at 30 °C and 250 μmol m^− 2^ s^− 1^ intense luminosity or at 16 °C and intense luminosity (50 μmol m^− 2^ s^− 1^), respectively (Fig. 1e, f). The chlorophyll content in mature leaves of LA1526 and AC showed no significant difference regardless of growth situation (Fig. 1d, e, f). These results indicated that LA1526 exhibited the white virescent leaves that could be enhanced by low temperature and high light intensity.

### Chloroplast development was impaired due to the defects in photosynthesis-related proteins accumulation in *wv* mutant

**Fig. 2:**
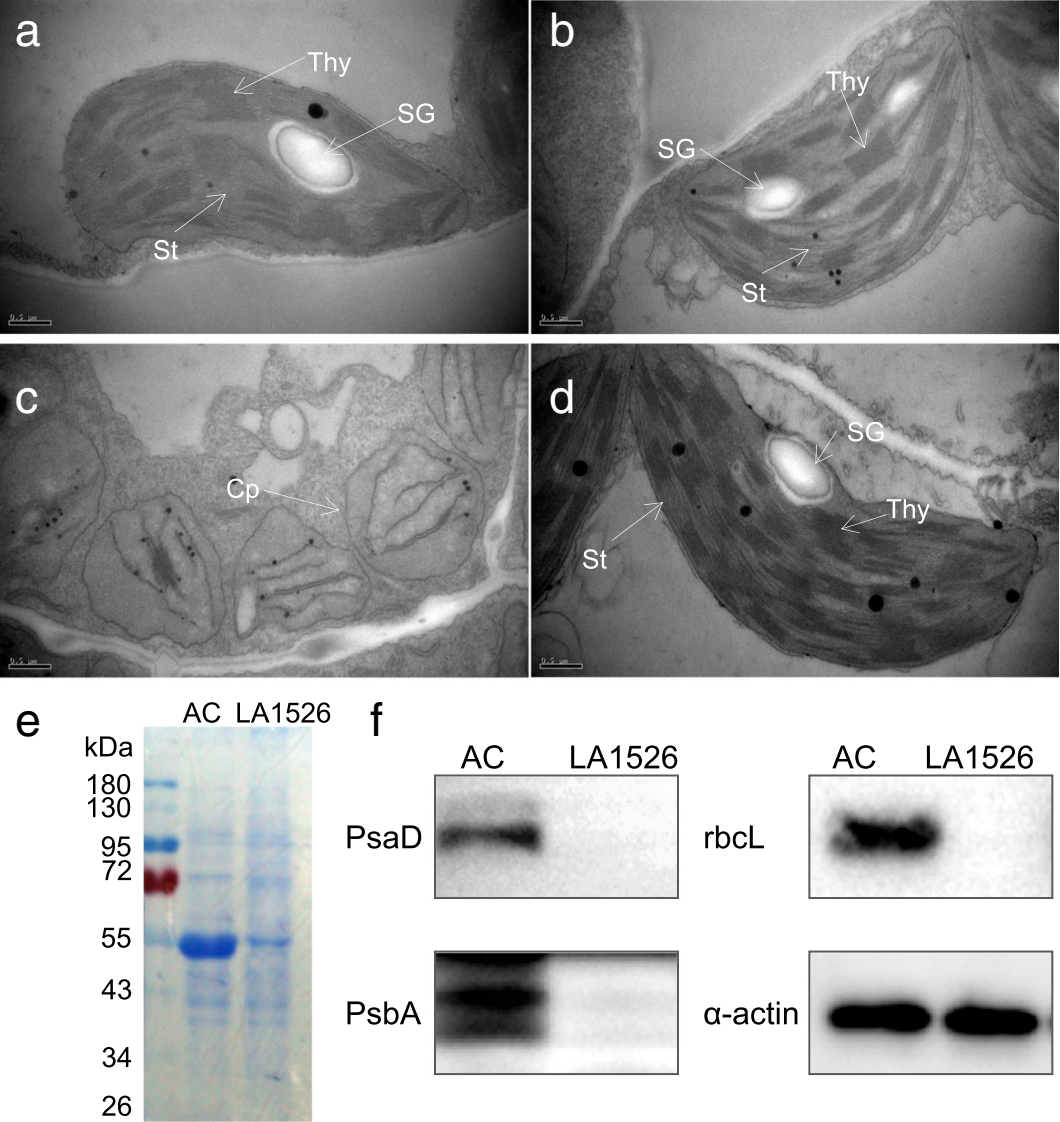
Ultrastructure of chloroplasts observation, protein analysis by SDS-PAGE, coomassie blue staining and immunoblot analysis in *wv* mutant and AC plants at 16 °C. **a**, **b** Chloroplasts in immature and mature leaves of AC plants, respectively. **c**, **d** Chloroplasts in immature and mature leaves of LA1526 plants, respectively. Cp, Chloroplast; St, Stroma; SG, starch grain; Thy, Thylakoid. **e** Protein analysis by SDS-PAGE and detection by coomassie blue staining. **f** Immunoblot analysis of chloroplast proteins in *wv* mutant. These proteins were PsaD (the photosystem I subunits); PsbA (photosystem II reaction center subunit); rbcL (the large subunit of Rubisco enzyme). The anti-actin served as a loading control. Magnifications of **a**, **b**, **c** and **d** are the same. Bar = 0.5 μm

Chloroplast development has an important impact on chlorophyll content, the defect of which impaired chlorophyll synthesis [28]. We observed the chloroplast ultrastructure by TEM and found that chloroplasts in AC immature, mature leaves, and LA1526 mature green leaves were crescent-shaped with well-developed stromal and stacked grana thylakoids (Fig. 2a, b, d). Additionally, starch grains were also observed in these plastids. However, the chloroplasts in LA1526 immature yellow leaves showed much smaller sizes and lacked organized membrane structures, including thylakoid membrane system and lamellar layer system of thylakoid. Moreover, chloroplasts contained no starch grains (Fig. 2c). These results demonstrated that *wv* possibly plays an important role in chloroplast biogenesis at an early stage. Analysis of total proteins by Coomassie blue staining assay after SDS-PAGE showed that many proteins, particularly the proteins of approximately 50 kDa in size, were decreased in the *wv* mutants (Fig. 2e). In addition, the accumulation of photosynthetic proteins in *wv* mutant was also detected by immunoblot analysis with the corresponding antibodies. The results showed that PsaD, PsbA, and rbcL, were sharply reduced in *wv* mutant (Fig. 2f; Additional file 1: Figure S1), suggesting that the accumulation of photosynthetic-related proteins was obviously inhibited in *wv* immature yellow leaves.

### *wv* was delimited to a 94 kb physical region on chromosome 2

**Fig. 3:**
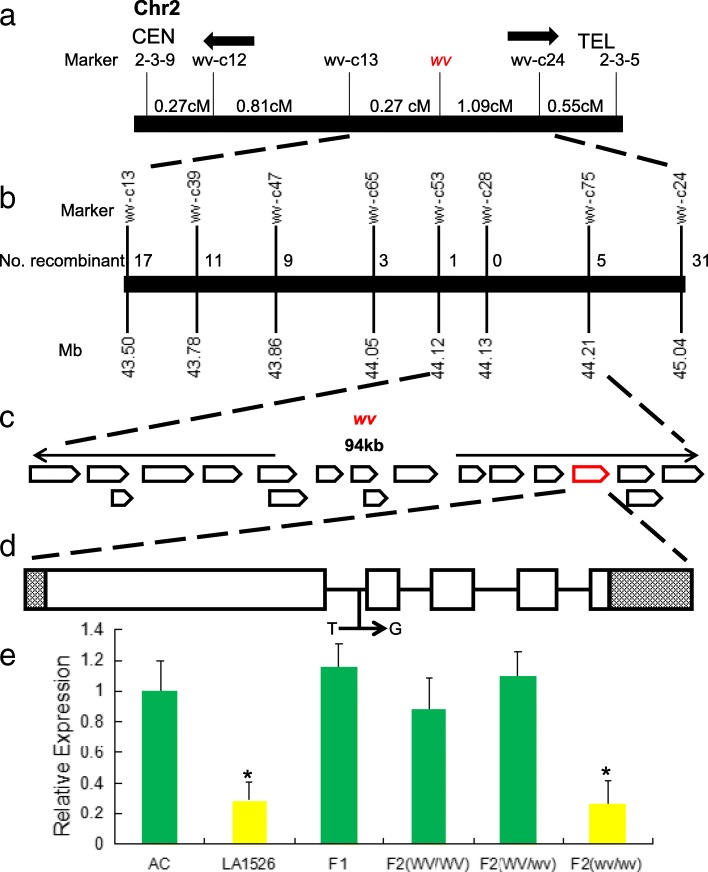
Map-based cloning of the *wv* locus. **a** Rough mapping of *wv* based on a mapping population of 186 plants. **b** Fine mapping of the *wv* locus based on 1602 F2 recessive individuals. **c** 18 expressed genes in the fine mapping of *wv*’s area. **d** Single base mutation in the first intron of the candidate *wv* gene, Solyc02g079730.2.1. Boxes denoted exons and lines indicate introns. Untranslated regions (UTRs) of gene were shown as boxes with hatched lines. **e** The expression level of Solyc02g079730.2.1 in LA1526, AC, F1 and F2 with three types of genotypes (WV/WV, WV/wv, wv/wv) in apical buds. The expression level in AC was set as 1.0 and other samples were calculated accordingly. Values were mean ± SD of three biological replicates. Asterisks indicated statistical significance at *P* < 0.01

Genetic analysis indicated that the white virescent phenotype in *wv* mutant LA1526 was controlled by a single recessive gene (Additional file 2: Table S4). The high-density molecular linkage map showed that *wv* was mapped to an interval flanked by TG14 and TG454 on the long arm of chromosome 2 [29]. These two markers were located at approximate 41.48 and 45.32 Mb on chromosome 2, respectively. To further determine the position of *wv* gene, we generated an F_2_ mapping population from the cross between LA1526 and IL2–3. Five markers, including 2–3-9, wv-c12, wv-c13, wv-c24, and 2–3-15 (Additional file 2: Table S5), were developed to screen 186 F_2_ recessive individuals. The linkage analysis showed that *wv* was delimited to an interval between wv-c13 and wv-c24, which were at a distance of approximate 0.27 and 1.09 cM, respectively (Fig. 3a). To further narrow the interval spanning the target locus, we conducted fine mapping based on 1602 F_2_ recessive plants. Forty-eight individuals displayed recombination events between two InDel markers, wv-c13 and wv-c24 (Fig. 3b). We thus developed six InDel molecular markers within this interval, including wv-c39, wv-c47, wc-c65, wv-c53, wv-c28, and wv-c75 (Additional file 2: Table S5). We investigated the genotypes of these recombinants by using these newly developed markers. We finally mapped *wv* gene in the region between wv-c53 and wv-c75, both of which were closely linked to *wv* with six recombination event identified. One marker wv-c28 was co-segregated with *wv* gene (Fig. 3b). We confirmed that *wv* gene was mapped to an approximately 94 kb fragment based on the tomato reference genome sequence (Fig. 3c).

### Solyc02g079730 was the candidate gene of *wv*

Based on the tomato genome annotation [ITAG Release 2.5 predicted coding sequence (CDS)], 18 putative ORFs were predicted in the target region (Fig. 3c). Analysis of the ~ 94 kb sequence by GENESCAN and FGENESH showed identical result. The best hits of these ORFs include receptor like protein kinases (ORF1- ORF13), an EF-hand type centrin, thioredoxin protein, U-box domain-containing protein, Flavoprotein wrbA and bHLH transcription factor (ORF14-ORF18), respectively (Additional file 2: Table S6). To further determine the possible candidate, we amplified and sequenced the genomic and cDNA sequences of these candidates from *wv* mutant LA1526, AC and M82. Sequence alignment showed that ORF15 (Solyc02g079730) in *wv* mutant LA1526 contains a single base mutation (T to G) (Fig. 3d; Additional file 1: Figure S2). We thus considered Solyc02g079730 as a good candidate for *wv* gene. Solyc02g079730 encoded a thioredoxin family protein, which is comprised of 365 amino acids. Interestingly, this nucleotide substitution did not result in amino acid substitution, implying that it possibly caused transcriptional change (Fig. 3d). We examined the expression of this gene in young leaves of AC, LA1526, F_1_ and F_2_ individuals from the cross IL2–3 × LA1526 (three genotypes, *WV*/*WV*, *WV*/*wv* and *wv*/*wv*) by using qRT-PCR. The expression of Solyc02g079730 was significantly repressed in LA1526 and F_2_ plants with *wv*/*wv* genotype (Fig. 3e). Overall, we inferred that the single base mutation possibly decreased the expression of Solyc02g079730 and led to white virescent leaves in LA1526.

### Mutation in Solyc02g079730 responsible for the white virescent phenotype in *wv* mutant

**Fig. 4:**
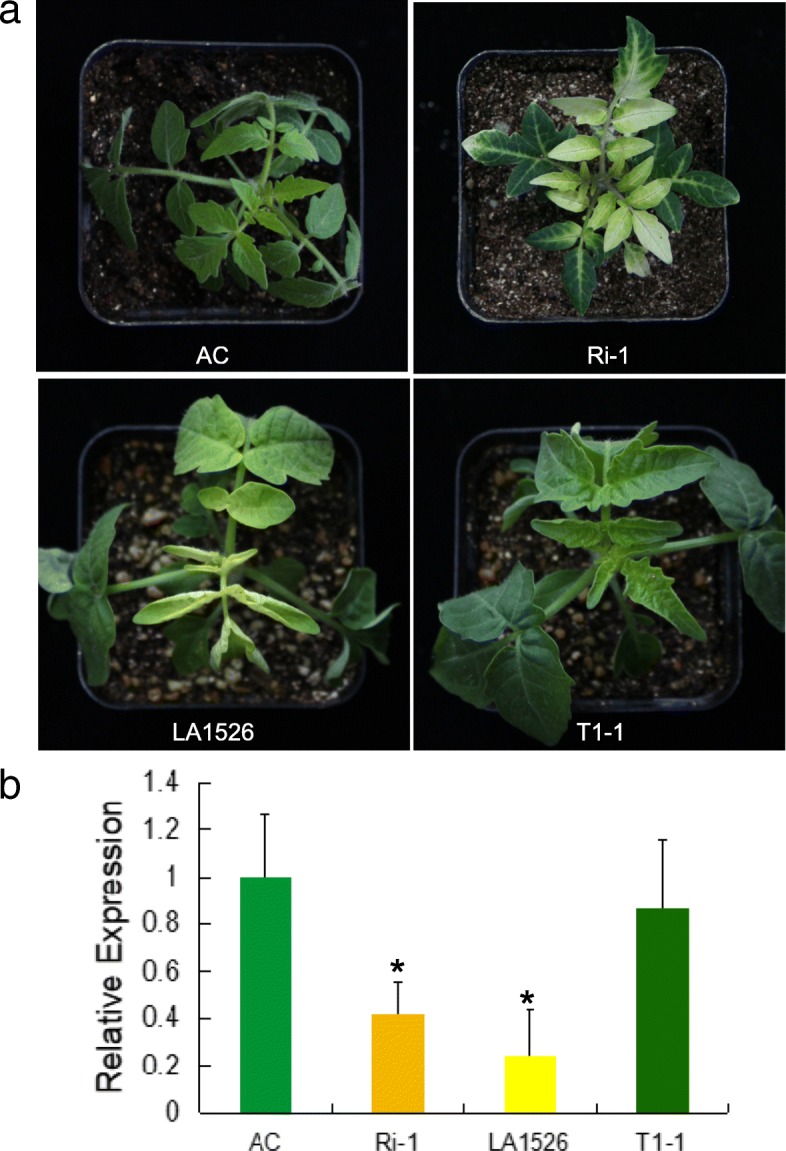
Transgenic analysis of *wv*. **a** Comparison of leaf blades color among AC plants, *WV* RNAi transgenic plants Ri-1, LA1526 and functional gene complementation transgenic T1 plants T1–1. **b** The expression level of *WV* in apical buds of AC, Ri-1, LA1526 and T1–1 at 16 °C. The expression level of *WV* in apical buds of AC plants at 16 °C with 250 μmolm^− 2^ s^− 1^ light intensity was provided as controls. Values were mean ± SD of three biological replicates. Asterisks indicated statistical significance at *P* < 0.01

**Fig. 5:**
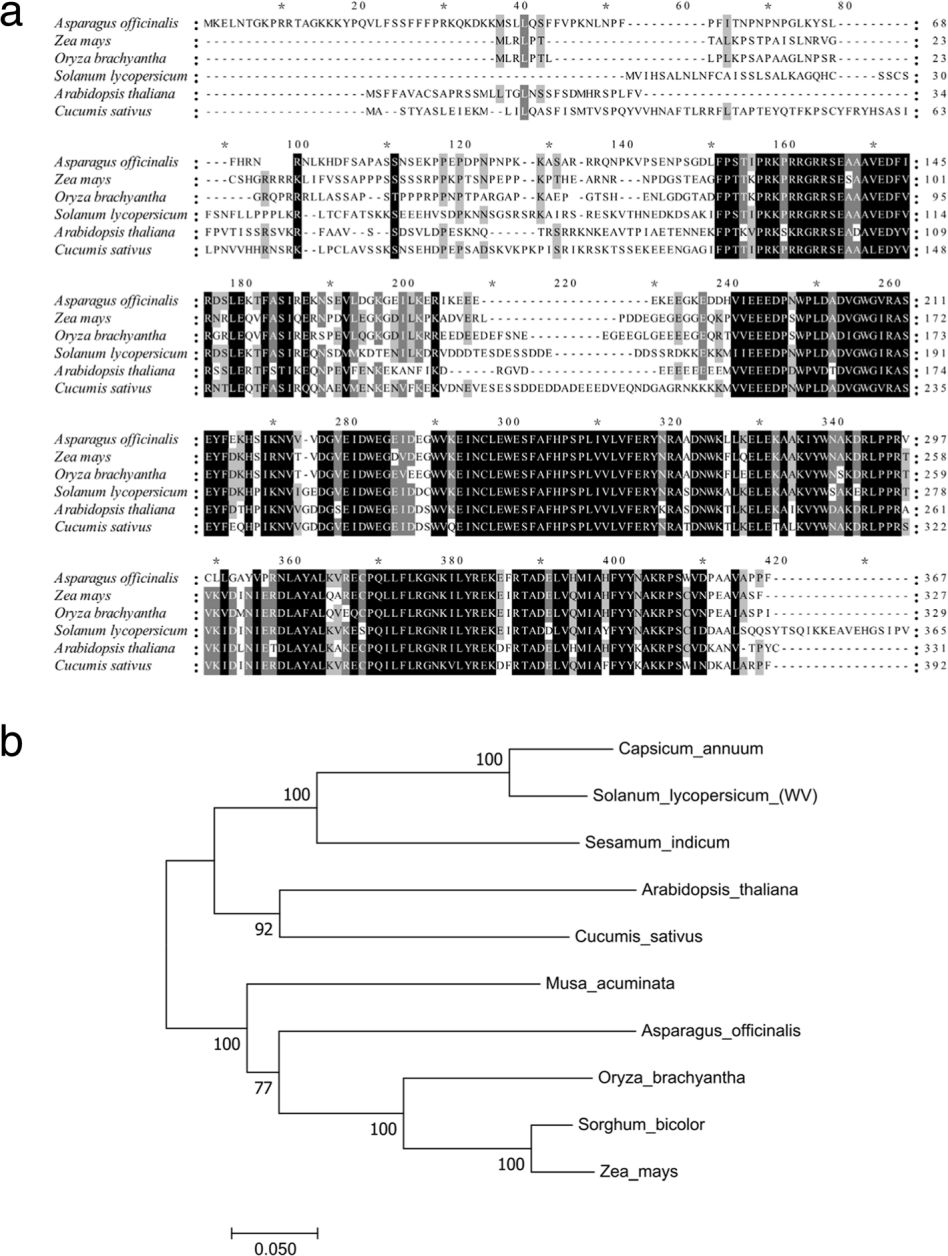
Sequence alignment and phylogenic analysis of *WV* and its homologs in different species. **a** Amino acid sequence alignment of *WV* with the five homologs. Amino acids fully or partially conserved were shaded black and gray, respectively. **b** Phylogenic tree of *WV* and homologs. Protein sequences were *Capsicum annuum* (XP_016560036.1), *Sesamum indicum* (XP_020550001.1), *Cucumis sativus* (XP_011651552.1), *Arabidopsis thaliana* (*SVR4*, AT4G28590.1), *Zea mays* (NP_001149252.1), *Oryza sativa* (XP_015643796.1), *Sorghum bicolor* (XP_021312876.1), *Asparagus officinalis* (ONK56897.1), *Hordeum vulgare* L. (BAJ87825.1), The rooted tree is based on a multiplesequence alignment generated with the program Mega6. Scale represented percentage substitution per site. Statistical support for the nodes was indicated

In order to investigate the biological function of Solyc02g079730, we prepared an expression construct by placing the full-length Solyc02g079730 from Ailsa Craig into pHellesgate 8 under control of its native promoter and introduced this construct into *wv* mutant LA1526 by stable transformation. We obtained three independent transgenic lines. qRT-PCR analysis showed that the expression of Solyc02g079730 was evidently up-regulated in transgenic plants (Fig. 4b; Additional file 1: Figure S3). As the sensitivity of white virescent phenotype to low temperature, we analyzed the phenotypic change of transgenic plants when growing at 16 °C. We found that the white virescent phenotype was rescued by the fragment from Ailsa Craig (Fig. 4a). Furthermore, we also generated RNAi transgenic lines (Ri) by using AC as recipient and found that they showed white virescent phenotype at 16 °C or 30 °C when Solyc02g079730 expression was decreased (Fig. 4a; Fig. 4b; Additional file 1: Figure S4). These results suggested that Solyc02g079730 is the candidate for *wv* gene. Through genomic and cDNA sequence comparison of *wv*, we determined that there were four exons and three introns. In addition, we conducted nucleotide and protein BLAST search against the National Center for Biotechnology Information and found that the homologs of *WV* gene widely exist in many different species, except for *Synechocystis sp*. PCC 6803 and *Clamydomonas reinhardtii*. Multi-alignment of the amino acid sequences encoded by homologues from other species indicated that they contained a highly conserved domain (Fig. 5a). WV showed a high amino acid sequence identity with a thioredoxin protein in cucumber (60%), *Arabidopsis* (63%), rice (67%), *Asparagus officinalis* (67%) and maize (69%), respectively., The homolog of WV in *Arabidopsis* had the highest homology and the same gene structure as SVR4/MRL7, which was involved in the regulation of early chloroplasts biogenesis [30, 31]. The phylogenetic tree demonstrated that these genes can be divided into two subgroups, in accordance with the biological taxonomy, monocots and dicots (Fig. 5b). These results suggested that an ancestral *WV* gene diverged prior to the split between monocots and dicots, and *WV* might be the functional ortholog of *SVR4*.

### Expression pattern of *WV*

**Fig. 6:**
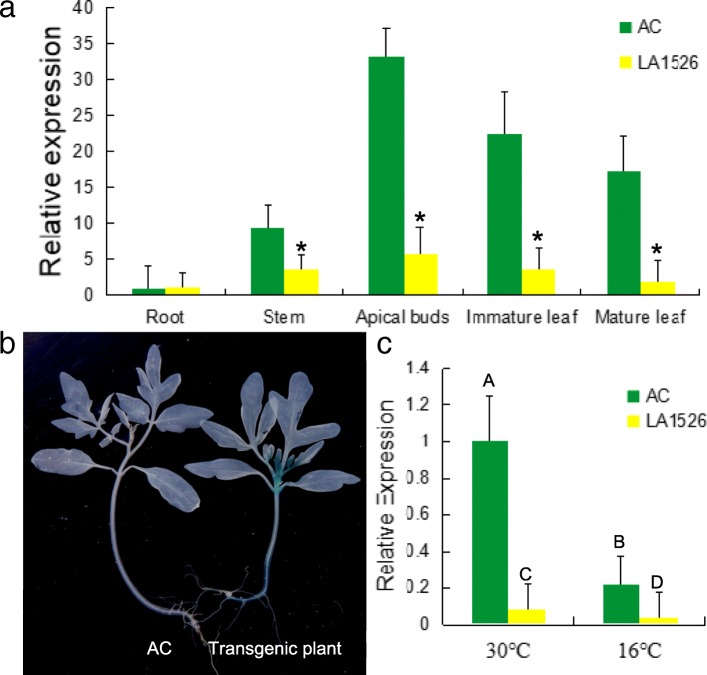
qRT-PCR analysis and histochemical GUS assay of *WV* gene. **a** The expression level of *wv* in different tissues of AC and LA1526. Values are mean ± SD of three biological replicates. Asterisks indicate statistical significance at *P* < 0.01. **b** GUS activity analysis of 2-week-old AC and transgenic plant. **c** The expression level of *WV* in immature leaves of AC and LA1526 plants at 16 °C and 30 °C with 250 μmolm^− 2^ s^− 1^ light intensity, respectively. The expression level of *WV* in apical buds of AC plants at 30 °C was provided as controls. Values were mean ± SD of three biological replicates

To examine the expression pattern of *WV* gene, we conducted qRT-PCR analysis with total RNA extracted from several tissues of AC and LA1526, including roots, stems, apical buds, immature leaves and mature leaves. *WV* was expressed in all these tested tissues, being highest in apical buds of AC plants (Fig. 6a). However, the expression of *WV* gene was dramatically decreased in several tissues of LA1526 compared to AC, such as stems, apical buds, immature leaves, and mature leaves (Fig. 6a). It has been suggested that some putative cis-elements in its promoter play important roles in the tissue-specific gene expression. Therefore, we detected cis-element of the promoter sequence of *WV* by using the Plant CARE and the PLACE program. We identified five types of putative cis-elements, including light response elements (3-AF1 binding site, ACE, Box 4, G-Box, Gap-box), MYB binding site (MBS), heat stress response element (HSE), defense and stress response element (TC-rich repeats), gibberellin-responsive element (GARE-motif). We further conducted *WV* promoter-driven GUS transformation to analyze the spatial expression pattern of *WV* gene. In accordance with our previous results, GUS was highly expressed in apical buds (Fig. 6b). Furthermore, we identified that the expression of *WV* was significantly down-regulated in both *wv* mutant and AC plants at 16 °C (Fig. 6c). Taken together, the expression pattern of *WV* is consistent with the target phenotype.

### *WV* affected the expression of chloroplast-encoded genes

**Fig. 7:**
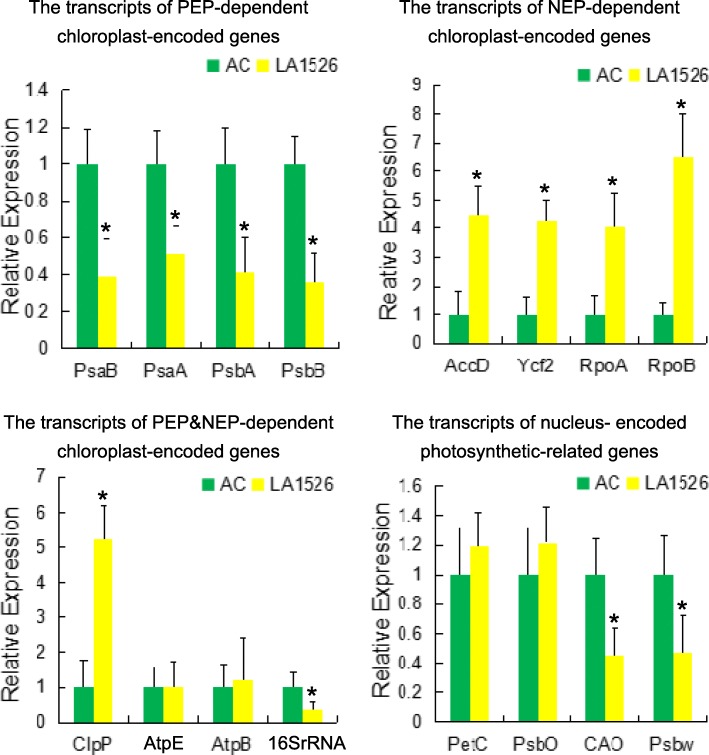
The expression of chloroplast and nucleus encoded photosynthetic-related genes in *wv* mutant and AC immature leaves by qRT-PCR. The chosen genes as representative PEP-dependent, NEP-dependent, PEP&NEP-dependent chloroplast-encoded genes and photosynthetic-related nucleus-encoded genes were used to analyze the impact of *wv* mutation on them. The expression level of each gene in AC plants was set as 1.0 and the expression level of each gene in *wv* mutant was calculated accordingly. Bars represented SDs of three biological replicates. Asterisks indicated statistical significance at *P* < 0.01

The expression of chloroplast-encoded genes was closely related to chloroplast developmental status, the expression of which was relied on collaboration between plastid-encoded polymerase (PEP) and nuclear-encoded polymerase (NEP) [32]. Therefore, we investigated the transcript profiles of various chloroplast-encoded genes in 4-week-old AC and *wv* mutant (Fig. 7). The results showed that the PEP-dependent genes, like *PsaB*, *PsaA*, *PsbA*, and *PsbB*, were decreased significantly in *wv* mutant compared to AC plants. In contrast, the expression level of NEP-dependent genes, like *AccD*, *Ycf2*, *RpoA*, and *RpoB*, were increased in *wv* mutant. Among PEP&NEP dependent genes, the abundance of *ClpP* transcripts in *wv* mutant was about 5 times as much as that in AC plants, whereas the abundance of *AtpE* and *AtpB* transcripts in *wv* mutant was the same as that in AC plants. In addition, the expression of *16SrRNA* was sharply reduced in *wv* mutant compared to AC plants. Furthermore, the expression level of nuclear-encoded genes destined for chloroplasts, such as *CAO* and *Psbw*, were much lower in *wv* mutant than that in AC plants. However, the transcripts of *PetC* and *PsbO* in *wv* mutant were almost identical to that in AC plants. These results indicated that the accumulation of many chloroplast-encoded genes was affected in *wv* mutants, which was probably the consequence of defective chloroplast biogenesis.

## Discussion

Plant leaves play essential roles in photosynthesis and then crop yield and quality. Chlorophyll biosynthesis and chloroplast formation are co-regulated by nuclear and plastid-encoded genes [33]. Chlorophyll-deficient mutants, like albino, variegated and virescent mutants, have been used to characterize the molecular mechanisms underlying the chloroplast development and chlorophyll synthesis in plants. Recently, many genes responsible for chlorophyll-deficient phenotypes have been identified in several plants, like *VAR2* in Arabidopsis and *V2* in rice [34, 35]. In this study, we identified a white virescent mutant. It has been suggested that many leaf-color mutants are sensitive to low temperature. For example, mutation in *Virescent 1* (*V1*) gene caused chlorotic leaves and sensitivity to low temperature [26]. Maize *virescent 16* mutant was unable to accumulate chlorophyll and exhibited chlorotic phenotype when grown at temperature lower than 25 °C [36]. Another maize temperature-sensitive mutant, *M-11*, was not able to accumulate chlorophyll below 17 °C [37]. Accordingly, we confirmed that *wv* mutant developed white leaves under low temperature condition, whereas exhibited green leaves under high temperature condition. Furthermore, we presented the evidence that the content of chlorophyll significantly decreased in the immature leaves of LA1526 at low temperature compared to that at high temperature. We thus speculated that the expression of *WV* gene responsible for *wv* phenotype may be sensitive to low temperature. In accordance with this reference, the expression of *WV* was significantly repressed in both *wv* mutant and AC plants at 16 °C compared to that at 30 °C. We further confirmed that the *wv* phenotype can be enhanced by high-light intensity, suggesting that *WV* may respond to light signal. It was further supported by the existence of temperature and light responsive cis-elements in the promoter of *WV*.

Since chlorophyll is synthesized in chloroplasts, impairment of chloroplast formation causes the reduction of chlorophyll content [38]. *MDA1* gene plays an important role in chloroplast formation, the mutation of which resulted in pigmentation reduction in Arabidopsis [39]. *SVR4* regulates early chloroplast biogenesis in Arabidopsis and *svr4* mutant exhibit the virescent phenotype [31]. In the present study, we observed the ultrastructure of chloroplasts through TEM and found that the chloroplasts in the young leaves of *wv* mutant lacked mature thylakoids and starch grains. Moreover, the expression of chloroplast-encoded PEP-dependent genes was obviously inhibited in immature leaves of *wv* mutant LA1526 compared to AC, including *PsaB*, *PsaA*, *PsbA* and *PsbB*. Consistently, a homolog of *WV* in Arabidopsis, *SVR4*, showed a similar regulatory role in the expression of chloroplast-encoded genes [30]. Interestingly, *WV* and *SVR4* had the same gene structure and conserved motif. Furthermore, the expression of chloroplast-encoded NEP dependent genes was significantly increased in immature leaves of *wv* mutant LA1526 compared to AC, such as *AccD*, *Ycf2*, *RpoA* and *RpoB.* And the expression of some nucleus-encoded photosynthetic genes was inhibited in immature leaves of LA1526, such as *CAO* and *PsbW*. Taken together, the data suggest that *WV* may be the functional ortholog of *SVR4*, which regulates chloroplast formation and chlorophyll accumulation through controlling the transcription of chloroplast-encoded PEP dependent genes. In addition, the up-regulated expression of NEP-dependent chloroplast genes and down-regulated expression of some nucleus-encoded photosynthetic genes were mainly influenced by the defects in the transcription of chloroplast-encoded PEP-dependent genes [30].

Based on the analysis of transgenic plants, we determined that the *wv* phenotype was caused by a single base mutation in *WV* gene, which encoded a thioredoxin family protein. Thioredoxins are small redox-active proteins, which are widely distributed among living organisms [40]. Previous studies showed that some of thioredoxins were involved in the regulation of chlorophyll metabolism and other processes in chloroplasts [41]. Therefore, knock-out mutations in some of these proteins usually impaired chloroplast formation and caused a severe reduction in chlorophyll accumulation [42]. Interestingly, we found that the mutation in the first intron of *WV* gene did not lead to amino acid substitution. We thus speculated that the white virescent leaves of *wv* mutant was possibly caused by the reduced level of *WV* transcripts. Accordingly, the expression of *WV* gene was significantly decreased in *wv* mutant LA1526. Furthermore, knockdown of *WV* gene through RNAi also resulted in similar phenotype to *wv* mutant, implying that the single base mutation inhibited the expression of *WV* gene, and then led to white virescent leaves. It has been reported that several plant introns probably contain enhancer elements and can enhance gene expression [43]. For example, the high-locule-number tomato fruit was possibly caused by an insertion in the first intron of *fas* gene, which repressed the expression of fas gene during early fruit development [44]. Additionally, small mutations in the second intron of the Arabidopsis *AG* gene could inhibit its expression [45]. Thus, it can be speculated that the first intron of *WV* gene possibly plays similar roles in the regulation of gene expression. Here, one pair of 11 bp reverse repeated sequences was found in the first intron of *WV*. This sequence-structure may be involved in intron-mediated enhancement. Coincidently, the nucleotide substitution occurred in the second repeat sequence, which possibly impaired the regulatory structure and inhibited the expression of *WV* gene. Moreover, the secondary structure of *wv* pre-mRNA may be altered by the single base mutation, which can reduce mRNA splicing efficiency and cause low abundance of *WV* gene transcripts. However, there is no more evidence to support this speculation. Therefore, it will be interesting to figure out the regulatory role of the first intron in the *WV* gene expression in the future.

## Conclusions

In this study, we identified a temperature- and light intensity-sensitive mutant, named as *wv* mutant. Based on positional cloning and transgenic analysis, we speculated that the single base mutation in the first intron of *WV* gene caused significant decreased expression of *WV* gene and blocked the differentiation of chloroplasts and synthesis of chlorophyll through inhibiting the transcription of chloroplast-encoded PEP dependent genes, which resulted in white virescent phenotype. Our results will help us to understand the regulatory role of thioredoxin protein in chloroplast differentiation. Moreover, further researches are necessary to elucidate the intron-mediated enhancement of gene expression during chloroplast formation and chlorophyll synthesis.

## Methods

### Plant materials and growth conditions

One introgression line (IL) 2–3 and *wv* mutant LA1526 were provided by the Tomato Genetics Resource Center (TGRC). IL2–3 contains the middle fragment of chromosome 2 of *S. pennellii* LA0716 genome in M82 background [46]. F_2_ mapping population segregated for the target phenotype was derived from a cross between IL2–3 and LA1526. All plants were grown under standard glasshouse conditions at 16 °C with 16 h day/8 h night cycle and 250 μmol m^− 2^ s^− 1^ intense luminosity. F_2_ individuals with white virescent leaves were used for mapping analysis. To detect whether *wv* mutant LA1526 was sensitive to temperature, plants were grown under the condition as follows: 16 h day/8 h night cycle, 24 °C, and 250 μmol m^− 2^ s^− 1^ intense luminosity. Two-week-old plants were divided into two groups, each of which was grown under 16 °C, and 30 °C conditions, respectively. Ailsa Craig (AC) plants as control were grown under the same conditions as described above. In addition, to detect the effect of light on *wv* mutant, we compared the second group with the third group, which was grown under 16 °C, 50 μmol m^− 2^ s^− 1^ intense luminosity.

### Chlorophyll and carotenoid content determination

Immature yellow leaves (the second leaves) and mature green leaves (the fourth leaves) were collected from 4-week-old LA1526 and AC plants and treated with 80% acetone in the dark. After grinding and high speed centrifugation, supernatant liquor containing pigments were immediately spectrophotometrically assayed at specific absorption coefficients using a microplate reader as described by a previous study [47].

### Transmission electron microscopy (TEM) observation

Samples (the second leaves and fourth leaves) collected from 4-week-old LA1526 and AC plants were cut into small pieces (~ 1 mm^2^) and inbubated in 3.5% (v/v) glutaraldehyde solution overnight. The fixed samples were then washed with 0.1 M phosphate buffer, post-fixed with 1% osmiophilic tetroxide, and dehydrated with a series of alcohol solutions, and then infiltrated and embedded with resin. The ultra-thin sections were prepared according to the methods described by Fan et al., 2016 [22]. Finally, the samples were observed and photographed using TEM (Hitachi H-7650, Tokyo, Japan) and Gatan 832 digital imaging system.

### Immunological detection of photosynthetic proteins

Total proteins were extracted from the top two leaves of 30-day-old AC plants and *wv* mutants LA1526 as described previous [30]. Approximately 1 g of each sample leaves was grinded into powder in liquid nitrogen, then lysed with ice-cold plant lysis buffer (50 mM Tris-HCl (pH 7.5), 150 mM NaCl, 0.5% TritonX-100) with complete protease inhibitor cocktail tablets (Roche, Basel, Switzerland), respectively. After incubating on ice for 30 min, the samples were centrifuged at 13000 g for 20 min at 4 °C to obtain total proteins in the supernatants. The supernatants transferred into a new tube were mixed well with equal amounts of 5x loading buffer and then boiled for 10 min.

Approximately 30 μg of total protein per sample were separated on 12% (m/v) sodium dodecyl sulfate PAGE (SDS-PAGE). After electrophoresis, the contents of the total proteins were detected by coomassie blue staining according to the manufacturer’s instructions. For immunodetection, the gels were blotted onto polyvinylidene fluoride (PVDF) membranes (Trans-Blot® Turbo TM Mini PVDF Transfer, Bio-Rad). After blocking with 5% skim milk for 2 h at room temperature, the membranes were washed twice in tris buffered saline with Tween 20 (TBST) and then incubated overnight at 4 °C with anti- PsaD, PsbA and rbcL rabbit primary antibody diluted 1000 times, respectively. Subsequently, the membranes were washed five times in TBST and incubated with secondary antibodies antibody against rabbit IgG at 1:3000 dilutions for 1 h at room temperature. Finally, the proteins were detected using chemiluminescence (ECL) method (Millipore, Burlington, MA). For all samples, the protein level of actin was used as a loading control which was detected with mouse anti-actin (AT3G12110; actin-11) primary antibody (manufacturer details) at 1:300 dilution. All polyclonal antibodies used in this study were obtained from ABclonal Technology. The common protocols and the manufacturers’ manuals for electrophoresis, semi-dry blotting and western detection using the ECL western blotting substrate were followed in this study.

### Positional cloning of *wv* gene

Plant genomic DNA was isolated from the young leaves of 3 to 4 weeks old seedlings by using a cetyltrimethyl ammonium bromide protocol [48]. The concentration of each DNA sample was determined by a NanoDrop 2000 spectrophotometer (Thermo Fisher Scientific) and adjusted to final 100 ng/μL. Primers for InDel markers were designed by using Primer 5.0 software based on the genomic sequences of M82 and LA0716 [49]. 186 F_2_ recessive individuals from the cross IL2–3 × LA1526 were initially used for rough mapping of *wv*. Then, a larger population of 1602 F_2_ recessive individuals was used for fine mapping of *wv* gene. Genotype data of each individual were adopted for linkage analysis by using the MAPMAKER/EXP 3.0 program [50].

### Molecular cloning and sequencing

We downloaded the genome sequence of Heinz 1706 in the target fragment based on the fine mapping results. The predicated genes in the target region were further analyzed by FGENESH (http://linux1.softberry.com/berry.phtml?topic=fgenesh&group=programs&subgroup=gfind) and GENESCAN (http://hollywood.mit.edu/GENSCAN.html). We amplified the candidate genes by using genomic DNA and cDNA of LA1526 and AC as templates. The primers are listed in Additional file 2: Table S1. PCR was performed in 20 μL mixture containing 100 ng of gDNA, 1 μL of 10 × Taq buffer, 0.2 μL of 10 mM dNTPs, 1 U of Taq DNA polymerase (Invitrogen, USA), and 2 μm of each primer. The amplification program was performed at 94 °C for 2 min, followed by 35 cycles at 94 °C for 30 s, 56 °C for 60 s, and 72 °C for 1 min, with a final extension at 72 °C for 5 min. PCR products were mixed with 2 μL of loading buffer and analyzed on a 2% agarose gel.

### Construction of expression vectors and generation of transgenic lines

To repress the expression of the Solyc02g079730.2.1, RNAi fragment was amplified with the primers listed in Additional file 2: Table S2 and inserted into the vector pHellsgate2 by using clonase BP reaction (Invitrogen). Furthermore, the full length genomic DNA of *WV* gene together with its 2.5 kb promoter sequence was cloned into pMV2 vector as described by a previous study [22]. The construct *proWV*::*WV* was transformed into *wv* mutant for functional complementation. In addition, we amplified an approximate 2.5 kb endogenous promoter fragment upstream of the start codon of *WV* gene. The fusion construct *proWV*::GUS was acquired by inserting the promoter fragment in front of the GUS coding region as described by Gao et al., 2017 [51]. The *proWV*::*WV* fusion construct was transformed into LA1526, whereas other constructs were transformed into AC mediated by *Agrobacterium tumefaciens* strain GV3101. The empty vectors of pHellsgate2 and pMV2 were also transformed as negative controls.

### Cis-element analysis of the *WV* gene promoter and histochemical GUS assay

An approximate 2.5 kb promoter fragment upstream the start codon of the *WV* gene was analyzed by using both NewPLACE (https://www.dna.affrc.go.jp/PLACE/?action=newplace) and plant CARE (http://bioinformatics.psb.ugent.be/webtools/plantcare/html/). Expression pattern of *WV* gene was reflected by GUS activity. In order to evaluate GUS activity, the 10 days old transgenic plants transformed with the pro*WV*::GUS fusion construct were immersed into a GUS staining solution and incubated overnight at 37 °C after vacuum infiltrating for 5 min. Then, the staining buffer was removed and chlorophyll was removed by incubating all samples in 70% ethanol. The GUS activity were observed and photographed by a microscope.

### Sequence and phylogenetic analysis

In order to identify homologs of *WV* gene in different species, pBLAST was conducted with *WV* amino acids at National Center for Biotechnology Information (NCBI) (https://www.ncbi.nlm.nih.gov/). Then, multiple sequence alignment was carried out by the Clustal Omega program (http://www.ebi.ac.uk/Tools/msa/clustalo/) and GENEDOC. Furthermore, the phylogenetic tree was constructed and tested by MEGA6.0 software based on the neighbor joining method.

### RNA extraction and qRT-PCR analysis

Total RNA was isolated using Trizol reagent (Invitrogen, USA) and treated by DNase I to remove any genomic DNA contamination. cDNA was synthesized by using reverse transcriptase (Toyobo, Japan) according to the manufacturer’s protocol. And the quality of cDNA was detected by PCR using Ef1a primers as follows: Ef1a forward (5′-GGCCACAGGGATTTCATCAAG-3′) and reverse (5′-GTCCCTTGTACCAGTCGAGGTTG-3′). The concentration of each cDNA sample was adjusted to 100 ng/μL using a NanoDrop 2000 spectrophotometer (Thermo Fisher Scientific). qRT-PCR was performed in 10 μL reactions, which consisted of 5 μL TransStart Green qRT-PCR SuperMix (TransGen Biotech), 0.5 μL of each primer, and 4 μL of first-strand cDNAs. The PCR reactions were carried out on the Roche LightCycler 480 II. The program was described as follows: preheated at 94 °C for 3 min, followed by 40 cycles of amplification (94 °C for 20 s, 58 °C for 20 s, and 72 °C for 30 s), and stopped by an extension (72 °C for 10 min). Actin was used as the internal control to normalize the relative expression level of each gene. The primers of actin were listed as follows: actin forward (5′-GTCCTCTTCCAGCCATCCAT-3′) and actin reverse (5′-ACCACTGAGCACAATGTTACCG-3′). For all qRT-PCR experiments were conducted in triplicate. All qRT-PCR primers of genes related to chlorophyll biosynthesis and photosynthesis are listed in Additional file 2: Table S3.

## Additional files

Additional file 1: **Figure S1.** Immunoblot analysis of chloroplast proteins in *wv* mutant and AC plants. These proteins were PsaD (the photosystem I subunits); PsbA (photosystem II reaction center subunit; 39 kDa); rbcL (the large subunit of Rubisco enzyme). M represented marker. The α-actin was used as a loading control. **Figure S2.** Alignment of CDS and genomic sequences of *wv* from AC and LA1526. **Figure S3.** qRT-PCR analysis of complemented *WV* in transgenic plants. T1–1,T1–2 and T1–3 represented three independent functional complemented transgenic lines. The expression level of *WV* in apical buds of LA1526 plants at 16 °C with 250 μmolm^− 2^ s^− 1^ light intensity was provided as controls.Values were mean ± SD of three technical replicates. Asterisks indicated statistical significance at *P* < 0.01. **Figure S4.** qRT-PCR analysis of *Solyc02g079730.2.1* RNAi transgenic plants. The expression level of *Solyc02g079730.2.1* in AC plants was provided as controls. Ri-1, Ri-2 and Ri-3 represented three independent RNAi transgenic lines. Each value represented the mean ± SE of three replicates. Asterisks indicated statistical significance at *P* < 0.01. (DOC 3295 kb)

Additional file 2: **Table S1.** Primers for amplifying CDS and promoters of candidate genes. **Table S2.** Primers for *Solyc02g079730.2.1* fusion vectors construction. **Table S3.** Primers for qRT-PCR of chloroplast and nucleus encoded photosynthetic-related genes. **Table S4.** Genetic segregation analysis of *wv* mutants in different generations. **Table S5.** Details of the markers for definitive mapping of *wv*. **Table S6.** Predicted genes between marker wv-c53 and wv-c75. (DOC 131 kb)

## Abbreviations

AC: Ailsa Craig
Chl: Chlorophyll
Cp: Chloroplast
ECL: chemiluminescence
gDNA: genomic DNA
GluTR: Glutamyl tRNA reductase
GUS: Beta-Glucuronidase
IL: Introgression line
NCBI: National Center for Biotechnology Information
NEP: Nuclear-encoded RNA polymerase
ORF: Open reading frame
PEP: Plastid-encoded RNA polymerase
qRT-PCR: Quantitative real time polymerase chain reaction
RNAi: RNA interference
SDS-PAGE: sodium dodecyl sulfate PAGE
SG: Starch grain
St: Stroma
TBST: tris buffered saline with Tween 20
TEM: Transmission electron microscopy
TGRC: Tomato Genetics Resource Center
Thy: Thylakoid
wv: White virescent
