# Supplementary material for: The tomato WV gene encoding a thioredoxin protein is essential for chloroplast development at low temperature and high light intensity
Source: BMC Plant Biol. 2019 Jun 20;19:265. doi: 10.1186/s12870-019-1829-4 (PMC6585109; doi:10.1186/s12870-019-1829-4)
Supplement: Supplementary file 1 — Figure S1. Immunoblot analysis of chloroplast proteins in wv mutant and AC plants. These proteins were PsaD (the photosystem I subunits); PsbA (photosystem II reaction center subunit; 39 kDa); rbcL (the large subunit of Rubisco enzyme). M represented marker. The α-actin was used as a loading control. Figure S2. Alignment of CDS and genomic sequences of wv from AC and LA1526. Figure S3. qRT-PCR analysis of complemented WV in transgenic plants. T1–1,T1–2 and T1–3 represented three independent functional complemented transgenic lines. The expression level of WV in apical buds of LA1526 plants at 16 °C with 250 μmolm− 2 s− 1 light intensity was provided as controls.Values were mean ± SD of three technical replicates. Asterisks indicated statistical significance at P < 0.01. Figure S4. qRT-PCR analysis of Solyc02g079730.2.1 RNAi transgenic plants. The expression level of Solyc02g079730.2.1 in AC plants was provided as controls. Ri-1, Ri-2 and Ri-3 represented three independent RNAi transgenic lines. Each value represented the mean ± SE of three replicates. Asterisks indicated statistical significance at P < 0.01. (DOC 3295 kb) [file 12870_2019_1829_MOESM1_ESM.doc]

M AC LA1526

M AC LA1526


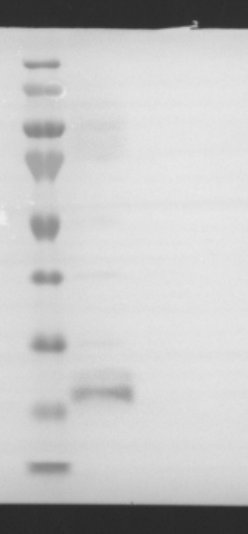

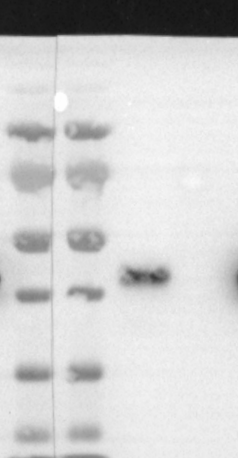


55KDa

26KDa

43KDa

rbcL

17KDa

PsaD

M AC LA1526 AC

M AC LA1526


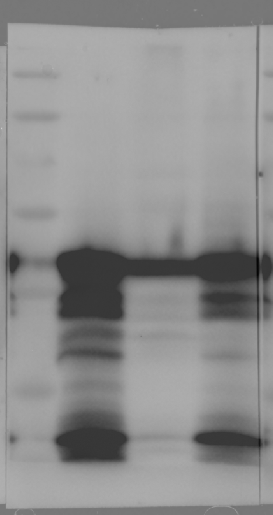

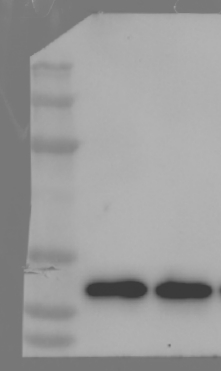


34KDa

43KDa

PsbA

34KDa

43KDa

α-actin

**Figure S1** Immunoblot analysis of chloroplast proteins in *wv* mutant and AC plants. These proteins were PsaD (the photosystem I subunits); PsbA (photosystem II reaction center subunit; 39kDa); rbcL (the large subunit of Rubisco enzyme). M represented marker. The α-actin was used as a loading control.


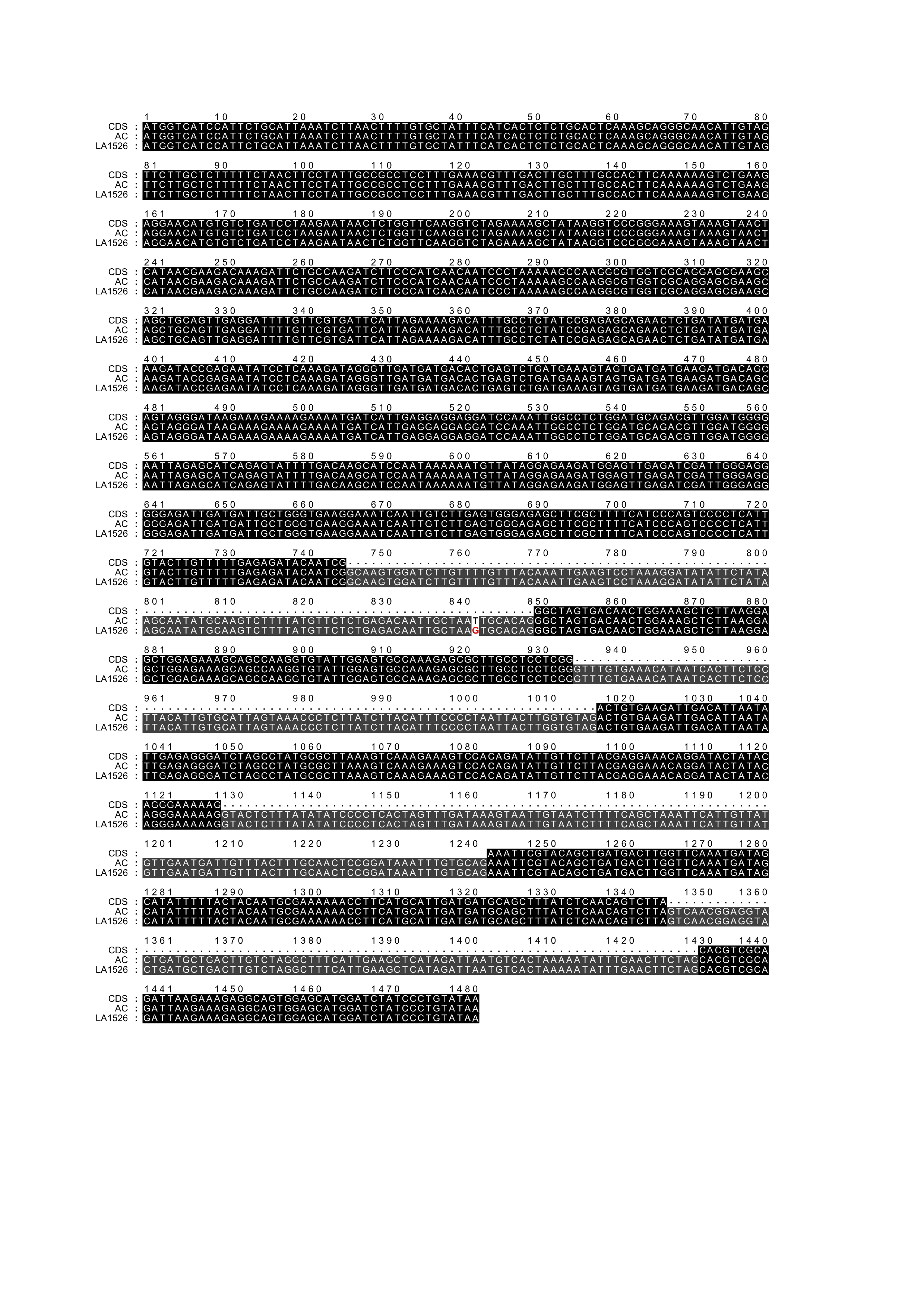


**Figure S2** Alignment of **CDS and genomic** sequences of *wv* from AC and LA1526.


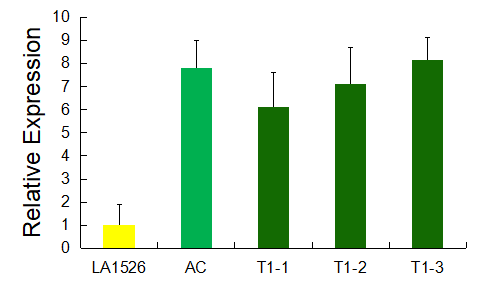


*

*

*

*

**Figure S3** qRT-PCR analysis of complemented *WV* in transgenic plants. T1-1,T1-2 and T1-3 represented three independent functional complemented transgenic lines. The expression level of *WV* in apical buds of LA1526 plants at 16 ℃ with 250 µmolm^-2^ s^-1^ light intensity was provided as controls.Values were mean ± SD of three technical replicates. Asterisks indicated statistical significance at P < 0.01.


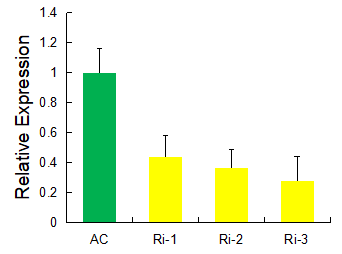


*

*

*

**Figure S4** qRT-PCR analysis of *Solyc02g079730.2.1* RNAi transgenic plants. The expression level of *Solyc02g079730.2.1* in AC plants was provided as controls. Ri-1, Ri-2 and Ri-3 represented three independent RNAi transgenic lines. Each value represented the mean ± SE of three replicates. Asterisks indicated statistical significance at P < 0.01.
