# Supplementary material for: The tomato WV gene encoding a thioredoxin protein is essential for chloroplast development at low temperature and high light intensity
Source: BMC Plant Biol. 2019 Jun 20;19:265. doi: 10.1186/s12870-019-1829-4 (PMC6585109; doi:10.1186/s12870-019-1829-4)
Supplement: Supplementary file 2 — Table S1. Primers for amplifying CDS and promoters of candidate genes. Table S2. Primers for Solyc02g079730.2.1 fusion vectors construction. Table S3. Primers for qRT-PCR of chloroplast and nucleus encoded photosynthetic-related genes. Table S4. Genetic segregation analysis of wv mutants in different generations. Table S5. Details of the markers for definitive mapping of wv. Table S6. Predicted genes between marker wv-c53 and wv-c75. (DOC 131 kb) [file 12870_2019_1829_MOESM2_ESM.doc]

Table S1 Primers for amplifying CDS and promoters of candidate genes

| Gene or gene promoter accession | | Primer sequence（5’-3’） | Product length（bp） |
| --- | --- | --- | --- |
| Solyc02g079590 | 1 | Fw: TCAAACAAGAGAACAAGTACACCAAC  Rv: AAAAGTTTCCTGTTAAGCATATGCA | 2902 |
|  | 2 | Fw: CCTAACCATATCTACCAATCACATCTACA  Rv: TTAGAAGGATATGGAAAAGGAATGA | 3248 |
| Solyc02g079600 | | Fw: TGCTCATTCCTGGAGACTGGTT  Rv: TGAGTGGGCTGCCATTTTGA | 3107 |
| Solyc02g079610 | | Fw: GTTGAGTATTTCGTATTTAAGCGCC  Rv: TGGCTGGGCTATTTGTTTCAC | 1773 |
| Solyc02g079620 | 1 | Fw: TTGGTTCAATGGCAATTATTGC  Rv: GATTAGGACACGATCTTCCGGTA | 3658 |
|  | 2 | Fw: ATTTGGGATGGTGCAGGAGTG  Rv: TGTATGGATTGGCTCAGATGGG | 3772 |
| Solyc02g079630 | | Fw: ATGTCAAATTGAGAGTTCATTTGC  Rv: AGAGACATTGGAAAATTTGGTGTC | 3652 |
| Solyc02g079640 | | Fw: TCTGTAGGCAAAATTTAATGCCTT  Rv: AGATTGCTCCTGATCCTTATGACT | 2996 |
| Solyc02g079650 | | Fw: ACTTCGGGGCAAGGGACG  Rv: GAAGCAACGGTTCCCTACCC | 2085 |
| Solyc02g079660 | | Fw: ATGAATAAGAGCATAGAGGACCACC  Rv: CAAAAGGGATTTCTCTGTCGC | 699 |
| Solyc02g079670 | | Fw: TGGCTGCTGTGAAGAAGAAGTG  Rv: TGCAAAGGGAAAGGGCAATA | 493 |
| Solyc02g079680 | | Fw: CCAAGAAGCCTAACAAGATTCTG  Rv: TTTACATCAGGGATTTTGGGTAAG | 532 |
| Solyc02g079690 | | Fw: TTATCAGCCGCCTTTTGGAC  Rv: GCAATAAGTCACACAGACTCAGTCG | 1896 |
| Solyc02g079700 | | Fw: CCCTCCCACACTTGAACAGAT  Rv: CTCTTATAATAGTGATTCCCCTCCA | 464 |
| Solyc02g079710 | | Fw: ACTTGCAAACACAATAGCCCTAC  Rv: TGGACACTTGAATGTTTGTTTGAT | 3246 |
| Solyc02g079720 | | Fw: TAATAGACAAGTCCGTAAGCTCGTCT  Rv: AGTACAGTATCCCTTAAAGGTGGACA | 496 |
| Solyc02g079720 promoter | | Fw: TTGAAATAGAAGGGCTCTTGCTGTC  Rv: CCCATCAGCTTAGAGTTCCTCTTTAAG | 3381 |
| Solyc02g079730 | | Fw:ACTGTAGCCAGAAGCAGACTTAGGA  Rv:TTAAGTAGATAAACAAGATCACCGCA | 2692 |
| Solyc02g079730 promoter | | Fw:TCAAATCAAAAGTAGCAAGAACAGC  Rv:ACATTGATGTTGAATTGTTGAGCAC | 2368 |
| Solyc02g079740 | | Fw: CATAAACCCTCTCTTACCTCCACAC  Rv: GGAAGCCTAAAAACTCACCTTCTTCT | 1327 |
| Solyc02g079740 promoter | | Fw: TTTGAACTTCTAGCACGTCGCA  Rv: CCGAAGGGCATGATTAGGG | 2874 |
| Solyc02g079750 | | Fw: TACGATAATGATGATGAGCGTGACA  Rv: CAATGTTCAAACTAGCTGTTGCTTC | 2977 |
| Solyc02g079750 promoter | | Fw: GGCCGCCTCCAATGTATCC  Rv: ACGAATCTTGCAGAGACTTAGGACC | 2433 |

Table S2 Primers for *Solyc02g079730.2.1* fusion vectors construction

| **Primers name** | **Primers sequences forward (5'-3')** | **Primers sequences reverse (5'-3')** |
| --- | --- | --- |
| RNAi | GGGGACAAGTTTGTACAAAAAAGCAGGCTTCAAAGATAGGGTTGATGAT | GGGGACCACTTTGTACAAGAAAGCTGGGTGCTCCTTAAGAGCTTTCCAG |
| Complementation | TGCATCCAACGCGTTGGGAGCTCTCAAATCAAAAGTAGCAAGAACAGC | TAAAGCAGGACTCTAGTGGTACCTTAAGTAGATAAACAAGATCACCGCA |
| Promoter-GUS | TGCATCCAACGCGTTGGGAGCTCTCAAATCAAAAGTAGCAAGAACAGC | TGCCTTCGCCATTCTAGACTCGAGCTTCCCTCGCTTAACTACTTCG |

Table S3 Primers for qRT-PCR of chloroplast and nucleus encoded photosynthetic-related genes

| **Abbreviation** | **annotation** | **Primers sequences forward (5'-3')** | **Primers sequences reverse (5'-3')** |
| --- | --- | --- | --- |
| PetC | Cytochrome b6-f complex iron-sulfur subunit | ATTTCATCGGTTTCACAAGCCT | AAGCAAGTGCACCCAATAAAAG |
| PsbO | Oxygen-evolving enhancer protein 1 of photosystem II | ACTGAAATCAAGGAATTGGCTC | ATTGGTTAGCAGTTCCAGTTCC |
| CAO | Chlorophyll a Oxygenase | AACCACGAAGAACGCTGAAT | TTGCTCAAAGCAATCAATCG |
| Psbw | a protein similar to photosystem II reaction center subunit W | TCAGCTCTATCCCGCCCTTC | CCATTGCTGTTGGGCTGAATG |
| PsbB | photosystem II CP47 chlorophyll apoprotein | GCTACTTTGAAATCCGATGGTG | TCCAAATTCGACTTGAGCATCT |
| PsaA | photosystem I P700 apoprotein A1 | CATTTCGGTCAACTCTCCATCAT | TAGTTATTCCAGATGCTCGCCA |
| PsbB | photosystem II CP47 chlorophyll apoprotein | GCTACTTTGAAATCCGATGGTG | TCCAAATTCGACTTGAGCATCT |
| AccD | acetyl-coenzyme A carboxylase carboxyl transferase | ACAAGCATTTGTGGGTTCAAT | AATACGGTCCTTATAAGGCTCCT |
| Ycf2 | hypothetical chloroplast RF2 | TTTCACGATCAAGGGTGTAATACTC | GGAAGATACAGGAGCGAAACAAT |
| RpoA | RNA polymerase alpha subunit | CGCCTTTATTATGGACGCTTTA | ATTCTTGAATGCCCGTTATGGT |
| RpoB | RNA polymerase beta subunit | GTAGGGATATGCAAGAACAAACAA | AATCTGATATTATGGTGCCGGT |
| ClpP | clp protease proteolytic subunit | ATTTGTCCTGGAAGCGATAGAA | ATAGATCTGCGTGAAATCCATG |
| AtpE | ATP synthase CF1 epsilon subunit | AATCCACAAGAAGCTCAGCAAA | TTATGAAATCGGATTGCTAGCC |
| AtpB | ATP synthase CF1 beta subunit | ACAATATCTTCCGTTTCGTCCA | TCTGCGGGTACATAAACTGCTT |
| 16ArRNA | 16S ribosomal RNA | AATAGCTTACCAAGGCGATGA | TTATTCCCCAGATACCGTCATT |

Table S4 Genetic segregation analysis of *wv* mutants in different generations

|  | No. of plants | | |  | Expected segregation |  |
| --- | --- | --- | --- | --- | --- | --- |
| Generation | Total | Green leaves | White virescent leaves |  | Ratio R: S | χ^2^(3:1) |
| P_1_ (IL2-3) | 25 | 25 |  |  |  |  |
| P_2_ (LA1526) | 25 |  | 25 |  |  |  |
| F_1_ | 60 | 60 |  |  |  |  |
| F_2_ | 620 | 474 | 146 |  | 3: 1 | 0.62<3.84 |

χ^2^_0.05_,_2_=3.84

Table S5 Details of the markers for definitive mapping of *wv*

| Marker name | Physical position（SL 2.50) | Primer sequence (5’–3’) | PCR product length (bp) |
| --- | --- | --- | --- |
| 2-3-9 | 42315068 | F:GTCCAAAGGTGCATCAATCC  R:AATATTTGTCGTGTCAACTCCC | 505 |
| wv-c12 | 42935622 | F:CTCACCAGATATTCCGTCAGGTAAT  R:TTGTATTCTTCACTTGCTTTCGCTA | 576 |
| wv-c13 | 43499917 | F:AACTTGCTACCTCTGGCACCTG  R:GACTTGGGTGTGACATGGGTATAGG | 564 |
| wv-c24 | 45035715 | F:ATGCACCTATTAGTGAAAGAACCCG  R:AACCGTTGCCATATTTGGATGTAGT | 333 |
| 2-3-15 | 45412650 | F:TGGATGTGGCCTAAGCAGA  R:GCAGCCTTTTGTTTTCATTG | 402 |
| wv-c39 | 43780722 | F:ATTCACCAAATCTTTTTGGAATGAA  R:TAAACATTTCCAAAGAAGGGCATAG | 280 |
| wv-c47 | 43857938 | F:TGTGAAAAGAAAGGATGCTTAGATT  R:AGTAACTACAAGACCATGACCTGCA | 553 |
| wc-c65 | 44052145 | F:TATGCCGAGATAAGTCTTTTCATCA  R:ATACAATATGTGTTGGCCCTCTCTG | 456 |
| wv-c53 | 44119152 | F:CCAATCACATCTACAATTTGCTTTC  R:TATCTCTTTGTGGACCCATTTATTG | 391 |
| wv-c28 | 44128046 | F:TCTGGATTCTAATACCATGATCAGG  R:TGTGTCAACTTCTTACCAACAAGCA | 542 |
| wv-c75 | 44213240 | F:TCAACTAATACATAATGATGGCGAA  R:ACTAGCGCACTAATTATTTTGGATG | 257 |

Table S6 Predicted genes between marker wv-c53 and wv-c75

| **ORF. no** | **Start** | **Stop** | **Length(bp)** | **Gene ID** | **Identifier** |
| --- | --- | --- | --- | --- | --- |
| ORF1 | 44116363 | 44122239 | 5,877 | Solyc02g079590.2 | Serine/threonine kinase receptor |
| ORF2 | 44123512 | 44126618 | 3,107 | Solyc02g079600.2 | Receptor-like protein kinase |
| ORF3 | 44125122 | 44126650 | 1,529 | Solyc02g079610.2 | Receptor serine/threonine kinase |
| ORF4 | 44129245 | 44136004 | 6,760 | Solyc02g079620.1 | Serine/threonine-protein kinase receptor |
| ORF5 | 44138888 | 44142243 | 3,356 | Solyc02g079630.1 | Receptor kinase |
| ORF6 | 44154777 | 44157667 | 2,891 | Solyc02g079640.2 | Serine/threonine-protein kinase receptor |
| ORF7 | 44156989 | 44158492 | 1,504 | Solyc02g079650.2 | Receptor protein kinase |
| ORF8 | 44158905 | 44159420 | 516 | Solyc02g079660.1 | Receptor-like protein kinase |
| ORF9 | 44159519 | 44159770 | 252 | Solyc02g079670.1 | Receptor-like kinase |
| ORF10 | 44159968 | 44160411 | 444 | Solyc02g079680.1 | Cysteine-rich receptor-like protein kinase 26 |
| ORF11 | 44161285 | 44162948 | 1,664 | Solyc02g079690.1 | S-locus receptor kinase (Fragment) |
| ORF12 | 44170249 | 44170506 | 258 | Solyc02g079700.1 | S-receptor kinase |
| ORF13 | 44170578 | 44173631 | 3,054 | Solyc02g079710.2 | Serine/threonine kinase receptor |
| ORF14 | 44180172 | 44180495 | 324 | Solyc02g079720.1 | Centrin |
| ORF15 | 44181929 | 44183799 | 1,871 | Solyc02g079730.2 | Thioredoxin family protein |
| ORF16 | 44186074 | 44187189 | 1,116 | Solyc02g079740.1 | U-box domain-containing protein |
| ORF17 | 44196835 | 44199442 | 2,608 | Solyc02g079750.2 | Flavoprotein wrbA |
| ORF18 | 44205177 | 44207837 | 2,661 | Solyc02g079760.2 | bHLH transcription factor-like protein |
